# CDK4/6 inhibition provides a potent adjunct to Her2-targeted therapies in preclinical breast cancer models

**DOI:** 10.18632/genesandcancer.24

**Published:** 2014-07

**Authors:** Agnieszka K. Witkiewicz, Derek Cox, Erik S. Knudsen

**Affiliations:** ^1^ Department of Pathology, Simmons Cancer Center, Dalls, TX; ^2^ Department of Pathology, UT Southwestern, Dallas, TX

**Keywords:** RB, HER2, Palbocicllb, CDK4, T-DM1

## Abstract

In spite of the efficacy of Her2-targeted therapies, recurrence and progression remain a challenge for treatment of Her2 positive breast cancer. CDK4/6 controls pathway downstream of Her2, Inhibition of these kinases could represent an important therapeutic approach to augment the effectiveness of standard therapies. In models of acquired resistance to Her2-targeted therapies, Cyclin D1 was inappropriately activated and CDK4/6 inhibition was effective at blocking proliferation by targeting this common pathway associated with resistance. These data were recapitulated in Her2 positive xenografts. Furthermore, in a series of 35 primary breast tumor explants, treatment with PD-0332991 resulted in a greater than 4-fold suppression of the Ki67. The effects of CDK4/6 inhibition were dependent on an intact RB-pathway, and consonantly, loss of RB and high-levels of p16 were associated with resistance to CDK4/6 inhibition. Combination studies illustrated that CDK4/6 inhibition is cooperative with multiple Her2-targeted agents and provides a complementary mechanism of action to T-DM1 to efficiently suppresses the proliferation of residual Her2-positive tumor cell populations that survive T-DM1. Together, these data indicate CDK4/6 is a viable therapeutic target that functions downstream of Her2, and tissue based markers are available to direct rational utilization of CDK4/6 inhibitors in combination with Her2-targeted agents.

## INTRODUCTION

Breast cancer is managed based on the presence of discrete markers that are measured clinically to direct patient care(1-7). The histological over-expression (3+) or amplification of the EGF receptor 2 (HER2) is observed in approximately 25% of breast cancers(8, 9). HER2 is a bona-fide breast cancer oncogene that will induce oncogenic phenotypes in cultured cells and drive mammary tumorigenesis in transgenic models (10, 11). In general, HER2 driven cancers are rapidly proliferative and prone to metastatic spread particularly to the brain and viscera(12). In addition, high levels of HER2 are known to compromise the activity of endocrine agents targeting the estrogen receptors(13). These combined biological features of HER2-positive breast cancer contribute to the relatively poorprognosis of this subtype of breast cancer.

Since the over-expression of HER2 is a tumor-specific event it has been the subject of intense therapeutic targeting(1, 8). HER2 functions as a dimeric binding partner to activate other members of the epidermal growth factor receptor (EGFR) family. These receptors signal through multiple pathways including ERK/MAPK, PI3K/AKT, and JAK/STAT to stimulate tumor cell proliferation and survival(8). HER2-positive breast cancers are typically addicted to this signaling, and are thus particularly sensitive to inhibition of this pathway. Agents, such as Lapatinib, Neratinib, and Afatanib are small molecules that bind to the tyrosine kinase domain of EGFR proteins and inhibit kinase activity to antagonize the activity of HER2 (8). In contrast, Trastuzumab is a humanized antibody that binds to HER2 and both limits HER2 signaling and induces an immune response against the tumor (14, 15). Several similar humanized antibodies have been developed (e.g. Pertuzumab), as have antibody drug conjugates (e.g. T-DM1). The antibody conjugate T-DM1, which was recently approved for late-stage HER2-positive disease, is Trastuzumab conjugated to the potent microtubule-poison metansine. In this fashion, T-DM1 provides selective delivery of metansine to the tumor cells(16, 17). Antibodies, conjugates, and small-molecules have all been shown to provide clinical benefit and have received approval for treatment of different clinical manifestations of HER2-poisitve breast cancer(18-20).

While HER2-targeted agents are effective, there are multiple mechanisms through which resistance can emerge(21-23). In the case of Trastuzumab, the epitope targeted by the antibody can be lost and shedding of receptor is also expected to limit the efficacy of this class of agent (24, 25). Similarly, there is evidence of selection to a state of HER2-negativity (23). In addition to these mechanisms that directly alter the behavior of the receptor, deregulation of intracellular signaling pathways (e.g. PTEN loss) have been described to limit the effectiveness of HER2 targeted therapies and are associated with the emergence of resistance(26). Due to the fact that many of the known resistance mechanisms can bypass the broad portfolio of HER2 targeted agents, there is intense interest in defining new therapeutic targets downstream from HER2.

The CDK4/6 kinase complexes are a particularly compelling target in HER2 positive breast cancer. CDK4 and CDK6 are kinases that are activated by D-type cyclins (27-29). They act downstream of HER2 and represent the point at which mitogenic signaling interfaces with the cell cycle machinery to promote proliferation(30, 31). Importantly, CDK4/6 are also downstream of the majority of processes driving resistance to HER2 targeted therapy. Deregulated CDK4/6 activity can induce mammary tumor development in mice, and conversely disruption of the activity will limit tumor development (32-34). Of primary relevance, the deletion of Cyclin D1 will limit the development and maintenance of tumors driven by HER2 (33, 34). This phenomenon is specific to HER2 and the dependence on Cyclin D1 is not conserved with other oncogenic drivers, such as MYC. In mouse models, it has also been shown that pharmaceutical CDK4/6 inhibition will antagonize HER2-driven mammary tumor growth (35).

Here we interrogated CDK4/6 pathway as a therapeutic target in multiple preclinical models. Specifically, we found that in models of acquired resistance to HER2 inhibition, cyclin D1 control is deregulated. Such models, xenografts, and primary human tumor tissue were all sensitive to CDK4/6 inhibition. Since CDK4/6 inhibitors are cytostatic it is critical to combine them with other agents to elicit durable disease control. We evaluated their action with multiple small molecule inhibitors and demonstrated a general additive therapeutic effect. In the case of T-DM1, we demonstrated that CDK4/6 inhibition has a complementary mode of action to suppress the proliferation of residual clones of disease. These studies suggest several rational approaches to the use of CDK4/6 inhibitors in the treatment of HER2-positive breast cancer that are being interrogated in clinical trials.

## RESULTS

### Deregulation of RB-pathway with acquired resistance to lapatanib

**Figure 1:**
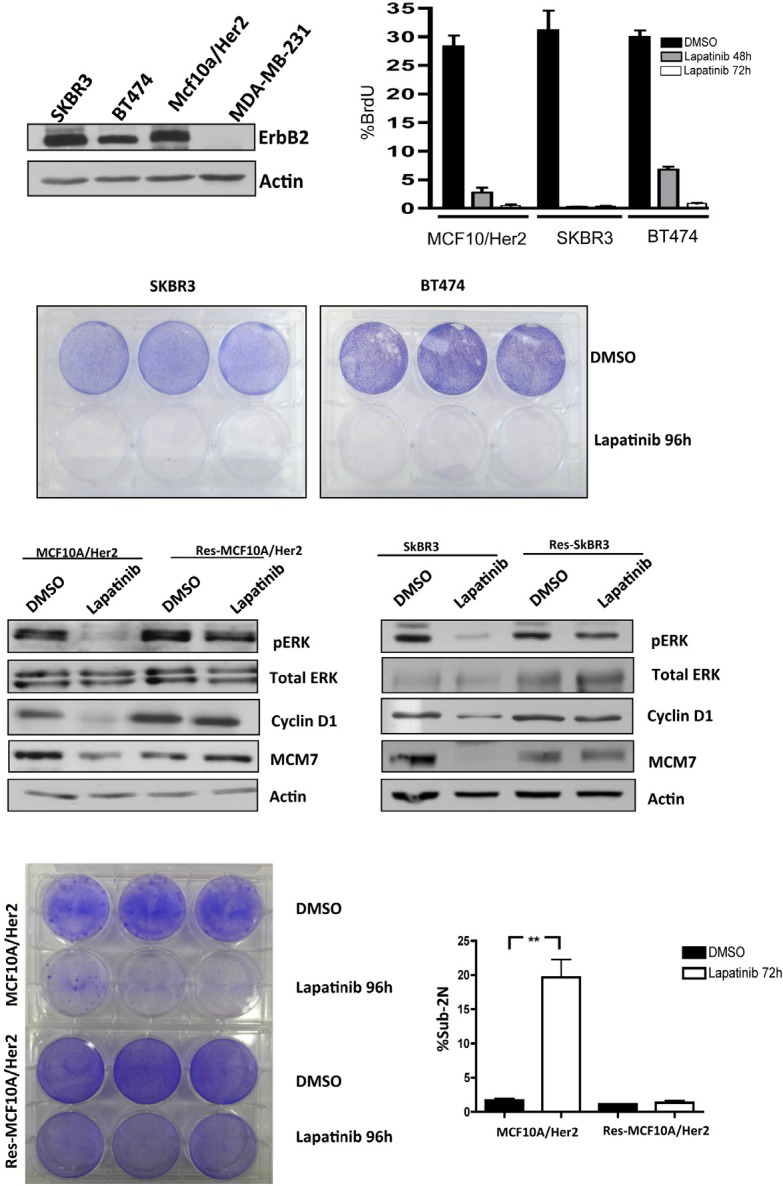
Acquired resistance to Lapatinib is associated with cell cycle uncoupling (A) (left panel) HER2 levels were detected in the indicated cell lines by immunoblotting. (right panel) Cells were treated with Lapatinib (1μM) for the indicated time and assayed for BrdU incorporation by flow cytometry. The effect of Lapatinib was significant under all conditions (p<0.01). (B) Cells were plated and treated with vehicle or Lapatinib (1μM) for 96 hours and plates were stained with crystal violet. (C) The indicated parental and resistant cell lines were evaluated for the biochemical response to Lapatinib (1μM). The indicated proteins were detected by immunoblotting. (D) (left panel) Cells were plated and treated with vehicle or Lapatinib (1μM) for 96 hours and plates were stained with crystal violet. (right panel) Sub-2N DNA content indicative of apoptotic cell death was determined by flow-cytometry (p<0.001).

For the analysis of HER2-positive breast cancer several cell models were employed (Figure 1A). Specifically, SKBR3 are HER2+/ER- and BT474 are HER2+/ER+. MCF10A cells were engineered to express levels of HER2 equivalent to the established cell lines, and MDA-MB231 cells are triple negative and used as a control. Each of the HER2+ models are sensitive to Lapatinib as shown by the suppression of BrdU incorporation (Figure 1A), and the loss of viability in crystal violet analysis (Figure 1B). In spite of the profound activity of Lapatinib, with continued treatment over 3-8 weeks resistant models of HER2/MCF10A and SKBR3 emerged. Such clones were resistant to the effects of Lapatinib on viability as determined by crystal violet analysis (Figure 1C) and were also refractory to the induction of cell death as measured by sub-2N DNA content (Figure 1C) annexin V staining and PARP cleavage (not shown). In analysis of signaling downstream from HER2, we observed that Lapatinib treatment routinely down-regulates ERK activity in both SKBR3 and HER2/MCF10A models, leading to the suppression of Cyclin D1 levels and the attenuation of E2F target genes such as MCM7 (Figure 1D). However, in the resistant clones, ERK activity was uncoupled from the action of Lapatinib and correspondingly cyclin D1 and MCM7 expression were no longer dependent on HER2 activity (Figure 1D). These data revealed that deregulation of the RB-pathway downstream from HER2 is a feature of acquired resistance.

### CDK4/6 inhibition has potent cytostatic activity in HER2 positive models

**Figure 2:**
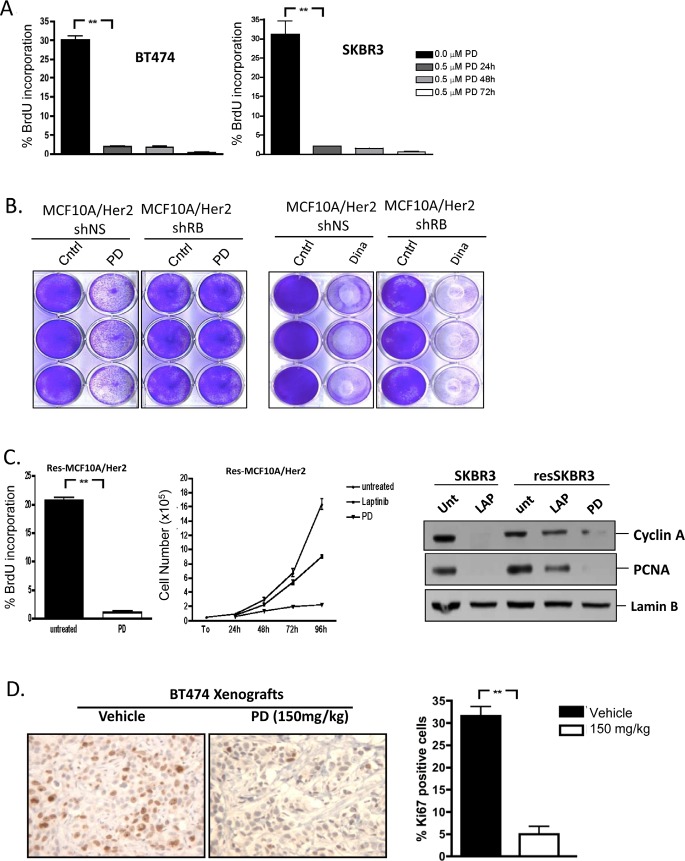
CDK4/6 inhibition has potent cytostatic effect in HER2-positive models (A) The indicated cells were treated with PD-0332991 (0.5 μM) for the indicated time and assayed for BrdU incorporation by flow cytometry. The effect of PD-0332991 was significant under all conditions (p<0.01). (B) The indicated cells were treated with PD-0332991 (1 μM) or Dinaciclib (1 μM) for 96 hours and plates were stained with crystal violet. (C) (left panels) Lapatinib resistant subcultures were treated with PD-0332991 (1μM) and BrdU incorporation determined by flow cytometry (p<0.01). The indicated cells were treated with vehicle, Lapatinib, or PD-0332991 and cell number determined daily by counting. (right panels) Cells were treated with the Lapatinib (1μM) or PD-0332991 (1 μM), and the indicated proteins were determined by immunoblotting. (D) BT474 xenografts were developed and mice were exposed to lactate buffer control or PD-0332991 (150 mg/kg) representative Ki67 staining and quantitation are shown (p<0.001).

Initially, the growth inhibitory activity of the CDK4/6 inhibitor PD-0332991 was evaluated in multiple HER2-positive models. As shown, treatment with PD-0332991 had a significant impact on BrdU incorporation in multiple models (Figure 2A). To determine if the effects on proliferation were specific to the RB-pathway, RB was stably knocked down by the use of shRNA. Control cell populations were clearly sensitive to PD-0332991 as demonstrated by the failure to proliferate (Figure 2B). In contrast, the RB-deficient populations proceeded to proliferate in the presence of PD-0332991. Importantly, this phenomenon was specific to CDK4/6 inhibitors, and the PAN-CDK inhibitor Dinaciclib killed all cells irrespective of the presence of RB (Figure 2B). In multiple models resistant to Lapatinib, PD-0332991 had potent cell cycle inhibitory activity (Figure 2C and not shown). Most importantly, PD-0332991 specifically suppressed long-term proliferation in cells resistant to the action of Lapatinib (Figure 2C). In this context, PD-0332991 functioned down-stream of the ostensible bypass mechanism in such models, and lead to the suppression of critical cell targets (e.g. PCNA and Cyclin A) in a fashion similar to that achieved with Lapatinib treatment of the parental cell models (Figure 2C). To determine if the anti-proliferative activity of PD-0332991 was actionable *in vivo*, mice were inoculated orthotopically with BT474 tumors (Figure 2D). When tumors were approximately 400 mm^3^, mice were treated with lactate buffer or PD-0332991 orally. Under these conditions, PD-0332991 had a highly significant effect on the proliferation of the tumor cells as determined by Ki67 staining (Figure 2D). Together these data indicate that CDK4/6 inhibition can have a profound impact on the proliferation of models driven by HER2 and derivatives that are resistant to HER2-targeted therapies.

### PD-0332991 has activity in HER2-positive primary breast tumor explants

**Figure 3:**
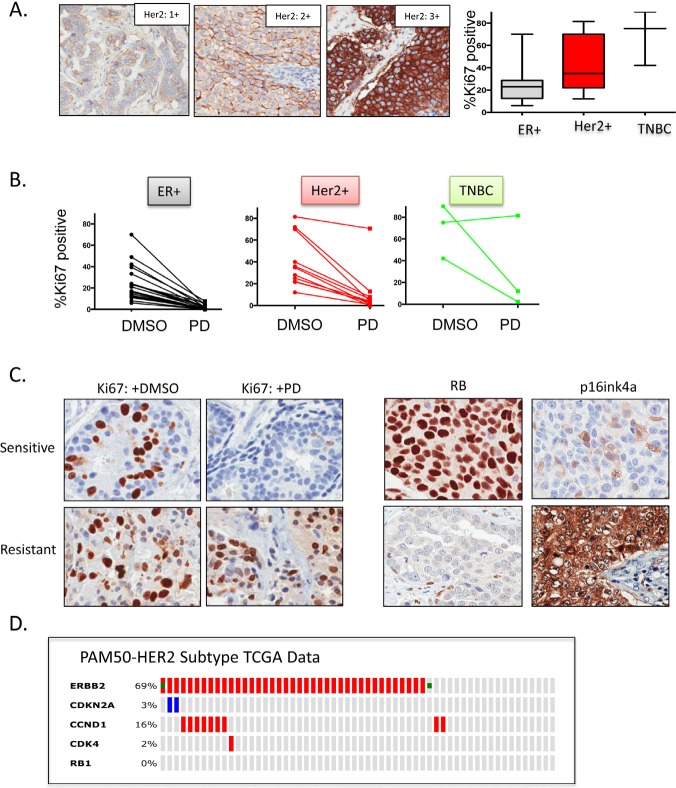
CDK4/6 inhibition is active in HER2-positive primary tumor explants (A) (left panel) representative staining of HER2 in the series of cases analyzed. (right panel) Ki67 labeling from different breast cancer explants, stratified by biomarker status (p=0.001 by Anova) (C) The Ki67 index for individual cases treated with vehicle or PD-0332991 (1 μM). (C) Representative Ki67 staining in sensitive and resistant explants. Staining of p16ink4a and RB identify pathway disruption in the resistant case. (D) Summary of TCGA genetic data for the HER2-subtype of breast cancer form CBIOPORTAL. Red denotes amplifications, Blue indicates homozygous deletion, Green indicates mutation.

Because no preclinical model adequately recapitulates the complexity/diversity of human disease, we performed an extended analysis of the sensitivity of primary tumor explants to PD-0332991 (Figure 3). We have previously published that PD-0332991 can suppress proliferation in such cultures using a small cohort(39). Here we expanded this cohort to include a total of 35 breast cancer cases, with an emphasis on HER2 3+ disease. Her2, ER and PR status was determined on all cases using clinical antibodies to unequivocally define the breast cancer subtype (representative staining for Her2, Figure 3A). By Ki67-staining, we found that the explant technology recapitulated the intrinsic proliferative difference between ER-positive, HER2-positive and triple negative breast cancers (Figure 3A). Importantly the vast majority of cases demonstrated a significant suppression of Ki67 (>4-fold reduction: 83%), with only 1 case of HER2 positive disease failing to respond to PD-0332991 (Figure 3B). In this case, loss of RB and superphysiological expression of p16ink4a was observed by immunohistochemical staining; which suggests that there are markers to define cases that will not respond to CDK4/6 inhibition (Figure 3C). Importantly, in the evaluation of clinical cases encompassed within the TCGA datasets, no cases were identified that exhibited RB loss within the HER2 intrinsic subtype of breast cancer (Figure 3D). These data suggest that the vast majority of HER2-positive tumors will have the capacity to respond to CDK4/6 inhibition in the clinic.

### Additive activity of CDK4/6 inhibition and small molecule inhibitors of HER2

**Figure 4:**
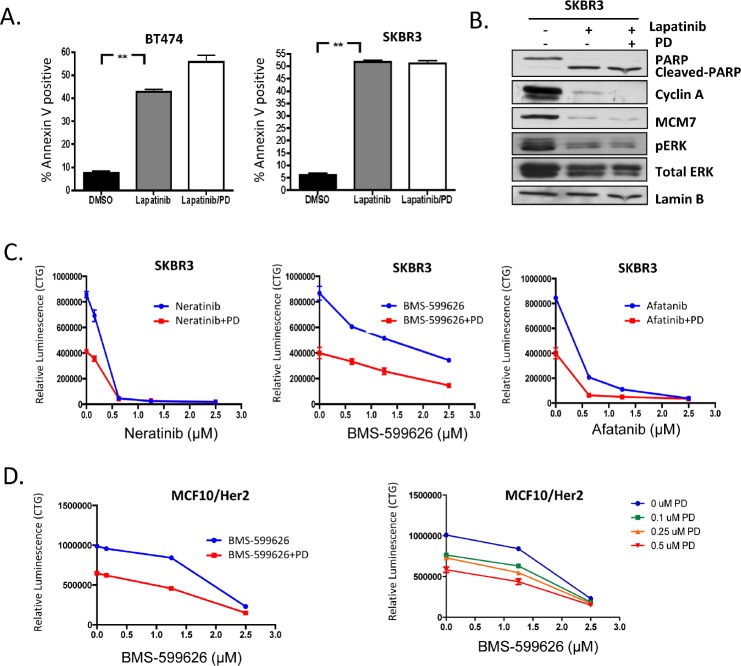
CDK4/6 has additive activity with HER2/EGRF kinase inhibitors (A) BT474 and SKBR3 cells were treated with Lapatinib concurrently with PD-0332991. Apoptosis was detected by Annexin V staining (p<0.01). (B) SKBR3 cells were treated with Lapatinib in the presence/absence of PD-0332991 and the indicated proteins were detected by immunoblotting. (C) SKBR3 cells were treated with Her2/EGFR inhibitors Neratinib, Afatanib, and BMS-599626 at increasing concentration in the presence/absence of 1 μM PD-0332991. Viability was measured by cell-titer-glow. (D) MCF10/Her2 cells were treated with increasing concentration of BMS-599626 at increasing concentration in the presence/absence of 1 μM PD-0332991. Viability was measured by cell-titer-glow. MCF10/Her2 cells were treated with increasing concentration of BMS-599626 at 0, 0.1, 0.25, and 0.5 μM PD-0332991. Viability was measured by cell-titer-glow.

Since HER2 targeted therapy is employed throughout the course of the treatment of HER2-positive breast cancer, even in the context of relapsed disease, we interrogated the functional interaction between CDK4/6 inhibition and HER2 inhibitors. As discussed above, Lapatinib will induce cell death in HER2-positive models, and in fact we observed that the Lapatinib induced cell death was not antagonized by CDK4/6 inhibition (Figure 4A). This finding contrasts, with the antagonism between CDK4/6 inhibitors and chemotherapy (35, 38). Mechanistically, Lapatinib yielded similar suppression of signaling through ERK and AKT in the presence or absence of PD-0322991 and equivalent suppression of down-stream targets (e.g. MCM7 and Cyclin A) (Figure 4B). To interrogate the overall relationship between CDK4/6 inhibition and Her2-targeted therapies, we investigated the dose dependent action with Neratinib, Afatinib, and BMS-599626. These data revealed that a fixed dose of PD-0332991 resulted in potent suppression of HER2-positive models even with low levels of HER2-targeted agent, and there was an additive relationship between the treatments (Figure 4C). These data were recapitulated in different models, and titration of both PD-0332991 and the Her2 targeted therapies revealed additive effects, even at relatively low concentration of PD-0332991 (Figure 4D). Together, these data suggest that combinations of HER2-targeted drugs with CDK4/6 inhibition could provide additive therapeutic benefit and prevent the proliferation of residual clones of cells that may escape either therapy.

### Distinct mechanisms of action between T-DM1 and PD-033291 in HER2-positive disease

**Figure 5:**
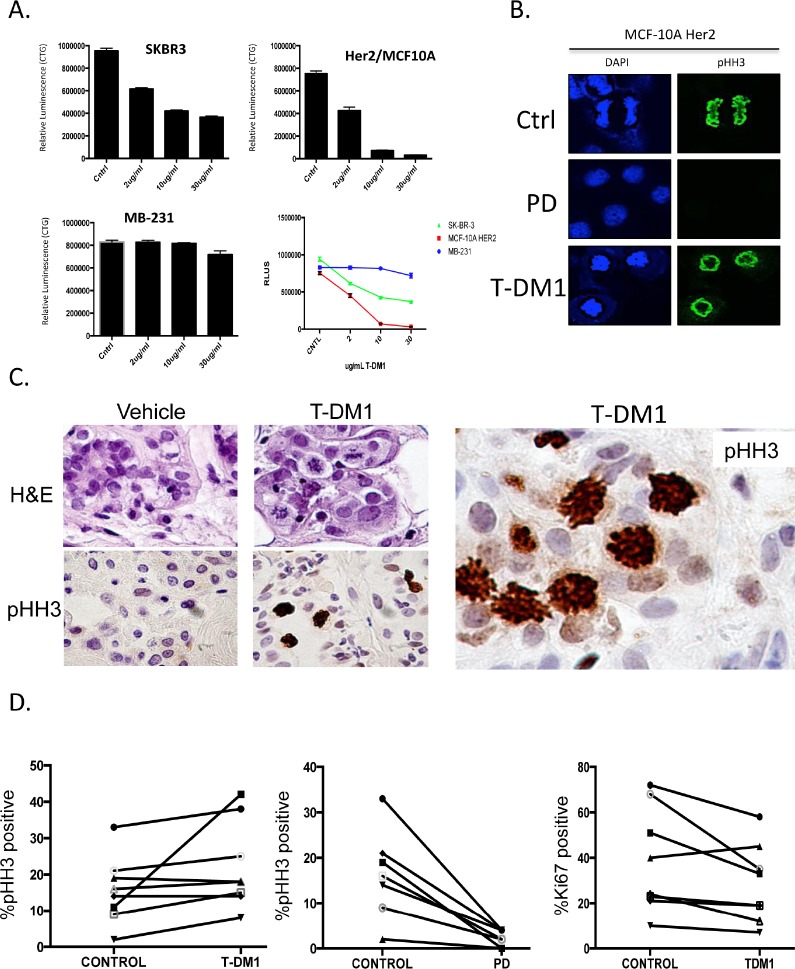
T-DM1 and CDK4/6 inhibitors have distinct mechanisms of action (A) The indicated cell lines were treated with T-DM1 and relative viability was determined by Cell Titer Glow. (B) The impact of T-DM1 or PD-0332991 on mitotic morphology was visualized by staining with PHH3. (C) Representative photomicrographs of primary tumor explants stained for PHH3 (D) Quantitation of PHH3 and Ki67 from primary tumor explants treated with T-DM1 or PD-0332991.

Currently, T-DM1 represents perhaps the most potent therapy for advanced HER2 positive breast cancer that has failed prior therapies(18, 41, 42). Therefore, we evaluated the mechanism of action and therapeutic interactions between T-DM1 and CDK4/6 inhibition. T-DM1 had a dose dependent effect on HER2-positive breast cancer models, while the triple negative breast cancer line MB-231 was refractory to the effects of the drug (Figure 5A). As expected, due to the presence of the microtubule poison, T-DM1 induced mitotic catastrophe (chromatin rings) that were readily visualized in treated cell population with phospho-Ser10 Histone H3 (Figure 5B). This overall response to treatment was observed in primary tumor explants, wherein multiple aberrant mitotic figures were detected in HER2-positive tumors (Figure 5C). Interestingly, in explants T-DM1 did not have a substantive effect on the overall levels Ki67 and phospho-Ser10 Histone H3 that are markers of proliferation. In contrast, phospho-Ser10 Histone H3 levels were substantially suppressed by CDK4/6 inhibition (Figure 5D).

**Figure 6:**
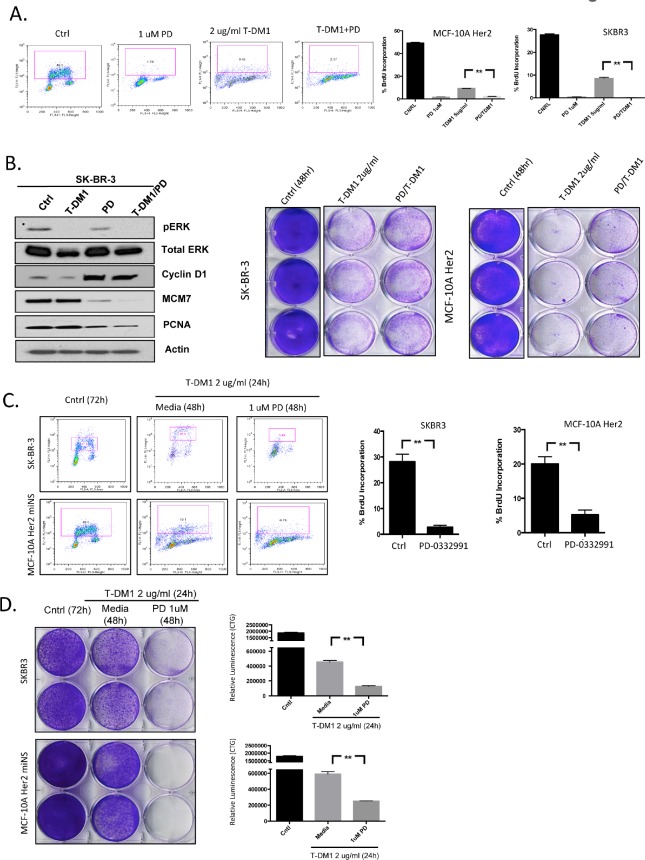
CDK4/6 inhibition prevents the emergence of residual clones following T-DM1 treatment (A) (left panels) Flowcytometric analysis of cells treated with the indicated drugs. (right panels) BrdU incorporation is quantified. (B) (left panels) Immunoblot analysis from cells treated with the indicated drugs. (right panels) Impact of concurrent treatment on T-DM1 mediated cytotoxicity was determined by crystal violet staining. (C) Flow cytometry following treatment with the indicated schedule and combinations of agents, BrdU incorporation is quantified (D) Crystal violet assessment (left panels) and Cell-titer-glow analysis (right panels) of single and sequential drug treatments as indicated.

To define mechanisms of action between drugs flow-cytometry and immunoblotting was performed, in concert with long-term viability assessment. We observed that T-DM1 induces a significant impact on cell cycle with suppression of cell populations throughout S-phase (Figure 6A). In contrast, PD-0332991 induces a G1 arrest (Figure 6A). Interestingly, the concurrent treatment of PD-0332991 with T-DM1 resulted in a hybrid profile indicative of the action of both agents; although CDK4/6 inhibition was dominant to T-DM1 in the suppression of BrdU incorporation (Figure 6A, right panel). Consistent with these findings, immunoblot analysis showed that although T-DM1 treatment suppressed the levels of ERK phosphorylation, it had a relatively modest effect on E2F-regulated genes such as MCM7 and PCNA (Figure 6B). These data indicate that T-DM1 and CDK4/6 inhibitors have highly distinct mechanisms of action, and could yield cooperative effects in terms of disease control. Under these conditions, T-DM1 had a profound effect on viability, and co-addition of PD-0332991 had a relatively modest effect related to the T-DM1 mediated cytotoxicity (Figures 6B, right panels). Although T-DM1 is a potent cytotoxic agent, residual cells survived, and within 48 hours these residual cells began to re-enter the cell cycle (Figure 6C). This finding suggested that sequential treatment CDK4/6 inhibition could suppress the proliferation of residual/resistant disease. By flow cytometry PD-0332991 clearly limited the ability of such residual cells to enter the cell cycle (Figure 6C). Correspondingly, post T-DM1 treatment CDK4/6 inhibition blocked the outgrowth of clones over 10 days (Figure 6D), and led to more pronounced long-term suppression of viable cells (Figure 6D). Together, these data support the concept that T-DM1 and CDK4/6 inhibition could be deployed in a sequential fashion.

## DISCUSSION

Her2-targeted therapies remain the mainstay for the treatment of Her2-positive breast cancer and are employed throughout the course of disease(8, 9). While such therapies are undeniably effective, many tumors recur and progress in spite of such agents. As a result, there is particular interest in utilizing targeted drugs to increase the durability of response and prevent the acquisition of resistance. Most resistance mechanisms identified for HER2-targeted therapies represent alterations to the receptor or intracellular signaling cascades(23). Many of these events occur upstream of the cell cycle; therefore, targeting CDK4/6 which is downstream of multiple resistance mechanisms is rational. Furthermore, HER2 positive breast cancer demonstrates a particular reliance on CDK4/6, which has emerged from the study of mouse models (33-35). Therefore, here we interrogated the efficacy of CDK4/6 inhibitors in many conditions relevant to the treatment of HER2-positive breast cancer.

Multiple preclinical models have been developed that demonstrate acquired resistance to HER2-targeted agents. Analysis of these models have yielded important insights into the mechanisms through which resistance is manifest. In veritably all of these models deregulation at the level of the membrane HER2 or cytoplasmic signaling is associated with resistance. These essential findings have been re-affirmed by the analysis of clinical specimens (8, 23). *A priori* most of the mechanisms would be expected to be sensitive to CDK4/6 inhibition and in fact across a wide spectrum of models we observed that CDK4/6 inhibition was highly effective in the suppression of proliferation of HER2 positive models. The one exception encountered was a rare HER2-positive tumor that harbored loss of RB. In any clinical setting such tumors can be identified using compound analysis of RB and p16ink4a expression. These tumor suppressors exhibit a reciprocal relationship and IHC against both represent rigorous means to interrogate the RB-pathway (43). One mechanism of bypass to HER2 targeted therapies that could ostensibly compromise the effect of CDK4/6 inhibitor is the amplification of Cyclin E (44). It has been shown that over-expression of cyclin E can bypass the action of PD-0332991(45); therefore, tumors harboring amplification of cyclin E could potentially be resistant to both HER2 and CDK4/6 targeted agents. Interestingly, in analysis of TCGA data we did observe cyclin E2 amplification in ~10% of HER2 breast cancer cases (46). However, in the analysis of primary breast cancer all RB-positive cases were sensitive to PD-0332991.

Invariably, even in the relapsed setting, HER2-positive breast cancers will be treated with HER2-targeted agents. Therefore, we extensively evaluated the interaction between CDK4/6 inhibition and established HER2 inhibitors. These data indicated that CDK4/6 inhibition has a complementary mechanism of action and is effective in models that will not respond to such agents. Particularly, the CDK4/6 inhibitor potentiated the suppression of proliferation/cell viability with a wide-range of compounds. In particular, the potent cytostatic activity of PD-0332991 served to limit the number of surviving cells at limiting doses of the HER2-targeted agents. Analysis of the dose-relationships indicated that PD-0332991 has an additive effect in combination with HER2-targeted agents. This finding is important as there is clearly antagonism of PD-0332991 with other therapeutic agents that would limit the capacity for concurrent treatment(35). Thus, the preclinical data with the agents interrogated would strongly suggest that concurrent treatment with HER2-targeted agents and CDK4/6 inhibitors would prove to be effective.

For recurrent disease, T-DM1 has emerged as a predominant therapy for HER2-positive disease based on superiority over Lapatinib/Capecitabline(18). T-DM1 combines the action of trastuzumab with a potent microtubule poison. Interestingly, only a handful of preclinical studies have been published with T-DM1 (42, 47, 48). In our work, T-DM1 induces mitotic catastrophes as a reflection of the action of metansine. This effect was observed in cell culture and primary tumor explants, and is consistent with the work of others in xenografts(48). In spite of the significant cytotoxicity, we observe multiple residual cells that survive such treatments, even at relatively very high doses of T-DM1. In this setting, the subsequent fate of the T-DM1 treated cells is obviously important. In the clinic T-DM1 is delivered once every 21 days; therefore, if residual cells can proliferate this could select for acquired resistance. We did find that T-DM1 treated cells can re-enter the cell cycle and subsequently proliferate relatively rapidly. These findings agree with recently published work in xenograft models (48). Importantly, CDK4/6 inhibition could completely suppress the outgrowth of such colonies. Thus, these data indicate that CDK4/6 inhibition could be employed metronomically in concert with T-DM1 to prevent the outgrowth of tumor cells that survive the initial treatment. These preclinical data suggest potential efficacy of rationally combined treatments of Her2-positive breast cancer with CDK4/6 inhibition in concert with standard Her2-targeted therapies.

## MATERIALS AND METHODS

### Cell Culture

The established HER2-positive cell lines BT474 and SKBR3 cells were propagated in either DMEM containing 10% FBS supplemented with 100 U/ml penicillin/streptomycin and 2mM L-glutamine or RPMI containing 10% FBS supplemented with 100 U/ml penicillin/streptomycin and 2mM L-glutamine respectively at 37°C and 5% CO_2_. The HER2-MCF10A cells that are RB-proficient or deficient have been previously described(36), and were maintained in DMEM/F12 supplemented with 5% Horse Serum, 20ng/ml EGF, 10ug/ml insulin, 1ng/ml cholera toxin, 100ug/ml hydrocortisone, 100U/ml penicillin/streptomycin and 2mM L-glutamine. The development of resistant models was achieved by culturing Her2-positive models in 100 nM-1μM Lapatinib for 3-8 weeks. Pooled resistant populations were utilized for all subsequent experiments.

### Drug treatments

The HER2/EGFR inhibitors Lapatinib, Afatinib, BMS599626, Neratinib were obtained from SelleckChem, as were the CDK inhibitors Palbociclib and Dinaciclib. Drugs were dissolved in DMSO and utilized at 100 nM-2.5 μM as described for the individual experiments. PD-0332991 was dissolved in lactate buffer for animal treatment. Animals were treated at 150 mg/kg as we have previously reported (37, 38). T-DM1 was obtained as a pharmaceutical discard from the UT Southwestern Medical Center Pharmacy and utilized at a dose range of 2-30 μg/ml.

### Xenografts

BT474 orthotopic xenografts were developed in nude mice as has been previously published. When tumors reached ~400 mm3, mice were treated with saline or PD-0332991 as has been previously described. Tumors were excised and stained for Ki67 using methods we have previously reported (38).

### Primary tumor explants

The explant protocol was identical to that published previously (39). Cases were obtained through the UTSW Shared Tissue Resource. Tissue staining was performed as previously described(39).

### Immunoblot analysis

Immunoblotting was carried out with antibodies against: Santa Cruz Biotechnology: phospho-ERK(sc-7383), ERK 1(sc-94), Cyclin D1(Ab3), Mcm7(sc-9966), PCNA(sc-56), and Actin(sc-8432), ERBB2 (sc-284).

### Flow Cytometry

For flow cytometry, cells were incubated in BrdU labeling reagent (Invitrogen, Frederick, MD) for 1hr. Harvested cells were stained for 1hr with purified FITC-conjugated antibodies (BD Biosciences, San Jose, CA). Data were acquired on a FACScan fluorescence activated cell sorter (BD Biosciences) and analyzed using FlowJo software (Treestar Inc., Ashland, OR).

### Cell Viability Assay

Cell viability was tested using CELLTITER-GLO™ (Promega, Madison, WI) and were assessed using a luminescent plate reader, Synergy 2 (BioTek, Winooski, VT) and analyzed using Gen5 Plate Reader Software v2.03.1 (BioTek).

### Immunofluorescent Microscopy

Immunofluorecent microscopy was performed as previously described with antibodies against pHH3)and DAPI (Life Technologies) (36, 40).
