# Correction: CDK4/6 inhibition provides a potent adjunct to Her2-targeted therapies in preclinical breast cancer models

**DOI:** 10.18632/genesandcancer.211

**Published:** 2021-03-12

**Authors:** Agnieszka K. Witkiewicz, Derek Cox, Erik S. Knudsen

**Affiliations:** ^1^Department of Pathology, Simmons Cancer Center, Dallas, TX; ^2^Department of Pathology, UT Southwestern, Dallas, TX

Original article: Genes&Cancer. 2014;5(7-8):261-272. https://www.genesandcancer.com/article/24/pdf/

**PMCID**: PMC4162138 **PMID**: 25221644 **DOI**: 10.18632/genesandcancer.24

This article has been corrected: The authors agree that Figure 1C and 2C b-actin and lamin are duplicated and then mis-labeled. The authors declare that this correction does not change the results or conclusions of this paper. The authors sincerely apologize for this error.

**Figure 1:**
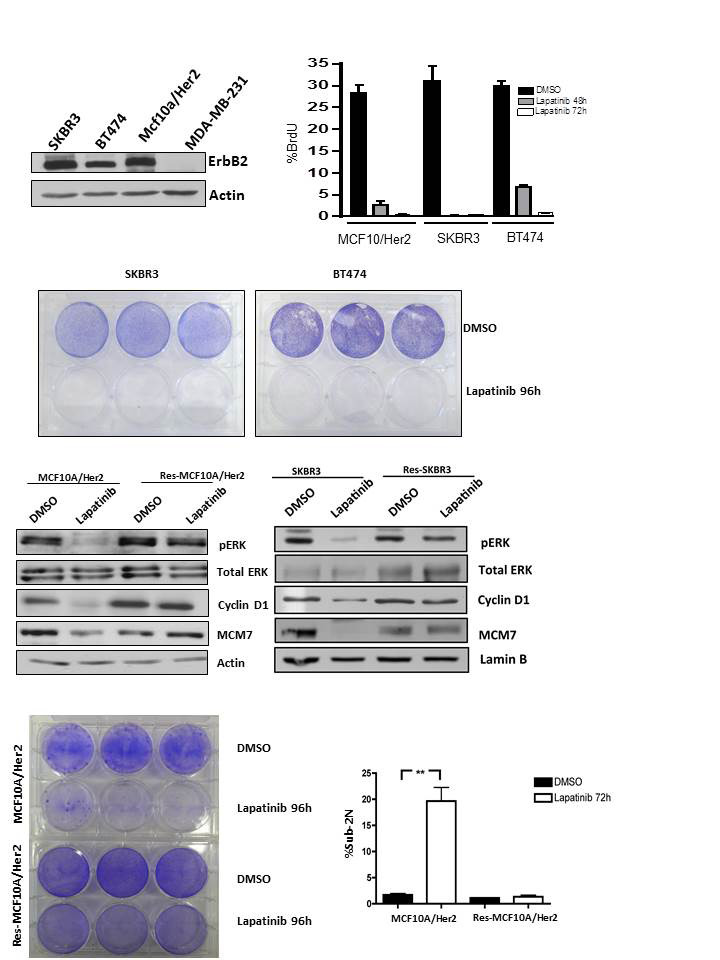
Acquired resistance to Lapatinib is associated with cell cycle uncoupling. **(A)** (left panel) HER2 levels were detected in the indicated cell lines by immunoblotting. (right panel) Cells were treated with Lapatinib (1µM) for the indicated time and assayed for BrdU incorporation by flow cytometry. The effect of Lapatinib was significant under all conditions (p<0.01). **(B)** Cells were plated and treated with vehicle or Lapatinib (1µM) for 96 hours and plates were stained with crystal violet. **(C)** The indicated parental and resistant cell lines were evaluated for the biochemical response to Lapatinib (1µM). The indicated proteins were detected by immunoblotting. **(D)** (left panel) Cells were plated and treated with vehicle or Lapatinib (1µM) for 96 hours and plates were stained with crystal violet. (right panel) Sub-2N DNA content indicative of apoptotic cell death was determined by flow-cytometry (p<0.001).

**Figure 2:**
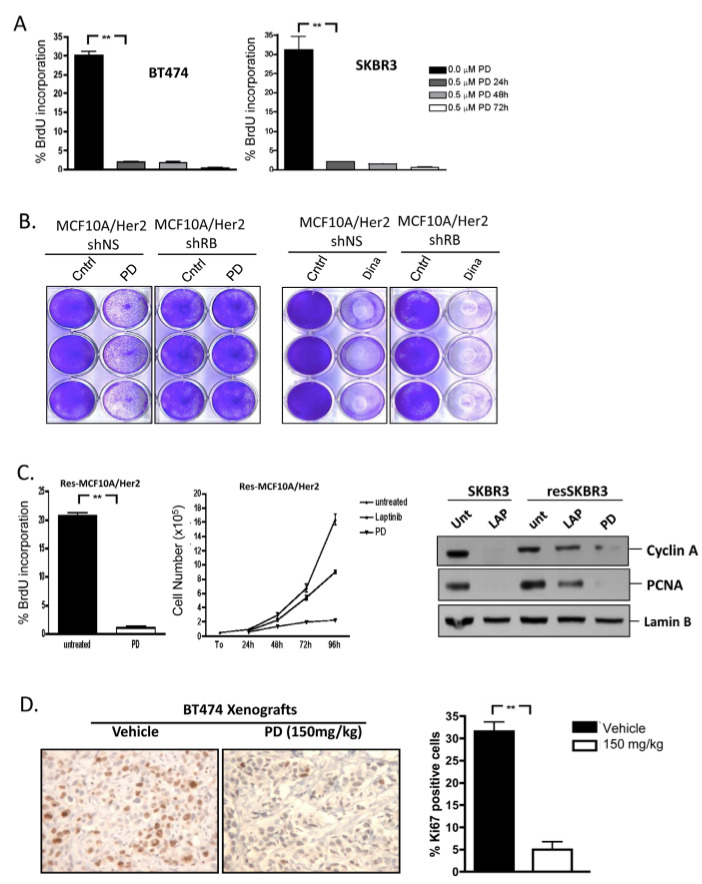
CDK4/6 inhibition has potent cytostatic effect in HER2-positive models. **(A)** The indicated cells were treated with PD-0332991 (0.5 µM) for the indicated time and assayed for BrdU incorporation by flow cytometry. The effect of PD-0332991 was significant under all conditions (p<0.01). **(B)** The indicated cells were treated with PD-0332991 (1 µM) or Dinaciclib (1 µM) for 96 hours and plates were stained with crystal violet. **(C)** (left panels) Lapatinib resistant subcultures were treated with PD-0332991 (1µM) and BrdU incorporation determined by flow cytometry (p<0.01). The indicated cells were treated wit vehicle, Lapatinib, or PD-0332991 and cell number determined daily by counting. (right panels) Cells were treated with the Lapatinib (1µM) or PD-0332991 (1 µM), and the indicated proteins were determined by immunoblotting. (D) BT474 xenografts were developed and mice were exposed to lactate buffer control or PD-0332991 (150 mg/kg) representative Ki67 staining and quantitation are shown (p<0.001).

